# Sm29, but Not Sm22.6 Retains its Ability to Induce a Protective Immune Response in Mice Previously Exposed to a *Schistosoma mansoni* Infection

**DOI:** 10.1371/journal.pntd.0003537

**Published:** 2015-02-27

**Authors:** Clarice Carvalho Alves, Neusa Araujo, Viviane Cristina Fernandes dos Santos, Flávia Bubula Couto, Natan R. G. Assis, Suellen B. Morais, Sérgio Costa Oliveira, Cristina Toscano Fonseca

**Affiliations:** 1 Laboratório de Esquistossomose, Centro de Pesquisas René Rachou, Fundação Oswaldo Cruz, Barro Preto, Belo Horizonte, Minas Gerais, Brasil; 2 Instituto de Ciências Biológicas, Universidade Federal de Minas Gerais, Pampulha, Belo Horizonte, Minas Gerais, Brasil; 3 Instituto Nacional de Ciências e Tecnologia em Doenças Tropicais (INCT-DT), CNPq, MCT, Salvador, Bahia, Brasil; The George Washington University Medical Center, UNITED STATES

## Abstract

**Background:**

A vaccine against schistosomiasis would have a great impact in disease elimination. Sm29 and Sm22.6 are two parasite tegument proteins which represent promising antigens to compose a vaccine. These antigens have been associated with resistance to infection and reinfection in individuals living in endemic area for the disease and induced partial protection when evaluated in immunization trials using naïve mice.

**Methodology/principals findings:**

In this study we evaluated rSm29 and rSm22.6 ability to induce protection in Balb/c mice that had been previously infected with *S*. *mansoni* and further treated with Praziquantel. Our results demonstrate that three doses of the vaccine containing rSm29 were necessary to elicit significant protection (26%–48%). Immunization of mice with rSm29 induced a significant production of IL-2, IFN-γ, IL-17, IL-4; significant production of specific antibodies; increased percentage of CD4+ central memory cells in comparison with infected and treated saline group and increased percentage of CD4+ effector memory cells in comparison with naïve Balb/c mice immunized with rSm29. On the other hand, although immunization with Sm22.6 induced a robust immune response, it failed to induce protection.

**Conclusion/significance:**

Our results demonstrate that rSm29 retains its ability to induce protection in previously infected animals, reinforcing its potential as a vaccine candidate.

## Author Summary

The development of a vaccine against schistosomiasis together with chemotherapy would have a great impact in the disease control and elimination. Sm29 and Sm22.6 are two promising antigens that have been associated with resistance to infection/reinfection in humans and also successfully induce protection in trials using C57BL/6 naïve mice. Despite the great results observed in C57BL/6 naïve mice, rSm29 and rSm22.6 ability to induce protection has never been assessed in mice previously exposed to the parasite antigens. In the case of schistosomiasis, this is an important assessment to be done, since the residents of endemic areas, the population mostly affected by the disease, are exposed to several infections through life. Here we evaluated these antigens in immunization trials using mice that had been submitted to a previous infection and treatment with Praziquantel. Both antigens induced a robust immune response triggering both cellular and humoral responses, but only rSm29 was able to induce a significant reduction on parasite burden and increased percentage of CD4+ memory cells. Our date reinforce Sm29 potential to compose an anti-schistosomiasis vaccine.

## Introduction

The development of a vaccine against schistosomiasis together with chemotherapy would have a great impact in the disease control and elimination. The ability of the parasite to evade the host immune system and its complex life cycle make the development of a vaccine against schistososmiasis a difficult task to achieve. But the presence of individuals naturally resistant to *Schistosoma mansoni* infection in endemic areas [1], the evidence of acquired resistance by constant infection and treatments over the time [2], and the high levels of protection induced by vaccination with irradiated cercariae suggest that developing a vaccine against the parasite is a feasible goal [3].

Many parasite antigens, especially the ones expressed on the parasite surface, have been studied as potential candidates for vaccine development [4,5]. Pre-clinical trials in the murine model represent an important step in anti-schistosomiasis vaccine development, and most of the antigens described as good candidates to be used in a vaccine formulation were evaluated in immunization protocols using naïve mice (Sm14, GST, Smp-80, TSP-2, Sm29, Sm22.6). Among the *S*. *mansoni* antigens, Sm29 and Sm22.6 are promising candidates. Sm22.6 or SmTAL-1 is a member of the *Schistosoma mansoni* Tegument-Allergen-Like (TAL) family [6]. Increased levels of IgE against Sm22.6 have been associated to resistance to reinfection in individuals living in endemic areas for schistosomiasis [6,7,8]. Also mice immunization with the recombinant form of Sm22.6 induced a significant decrease in parasite burden associated with increased levels of antibodies and mixed Type 1/Type 2 immune response [9]. Sm29 is a GPI-anchored parasite protein with unknown function [10]. High levels of IgG1 and IgG3 against the recombinant form of Sm29 were detected in individuals resistant to infection or reinfection [11]. Mice immunization with rSm29 elicited a significant antibody production and a Type 1 immune response, as well as a reduced parasite burden and pathology [12].

Besides the great results observed in pre-clinical trials in naïve mice using rSm29 and rSm22.6 as antigen, these antigens were never evaluated in pre-clinical trials using animals that had been previously exposed to parasite antigens. These evaluations are extremely important to determine antigen potential as a vaccine candidate, since the target population for the vaccine suffers several infections throughout life and is sensitized by parasite antigens *in utero*, which promotes a predominant Type 2 immunological profile [13,14,15].

In this context, we evaluated the ability of Sm22.6 and Sm29 recombinant proteins to induce protection with an immunization protocol using Balb/c mice previously infected with the LE strain of *S*. *mansoni* and treated with Praziquantel. We demonstrated that immunization of mice previously exposed to *S*. *mansoni* infection with rSm29 increased the levels of antibodies, IL-2, IFN-γ, IL-17 and IL-4 production and the percentage of CD4+ central memory T cells, and elicited a protection level ranging from 26% to 48%, while immunization with rSm22.6, despite inducing a robust immune response, failed to reduce worm burden.

## Methods

### Mice and parasites

Balb/c female mice aged 6–8 weeks were obtained from the Centro de Pesquisas René Rachou (CPqRR)—FIOCRUZ (Fundação Oswaldo Cruz) animal facility. *Schistosoma mansoni* cercariae (Sambon, 1907), LE strain, were maintained routinely on *Biomphalaria glabrata* (Say, 1818) snails at CPqRR and were obtained by exposing infected snails to light for 1–2 hours to induce shedding. All the protocols involving animal use in this study were licensed by the Ethics Committee of Animal Use (CEUA) of FIOCRUZ, under license number LW12/12.

### Antigens preparation

The recombinant Sm22.6 (rSm22.6) and Sm29 (rSm29) were produced and purified as previously described [9,11]. rSm22.6 and rSm29 concentrations were measured by BCA Protein Assay Kit (Thermo Scientific Pierce, Rockford, IL, USA).

### Infection, treatment and immunization protocol

**Fig 1:**
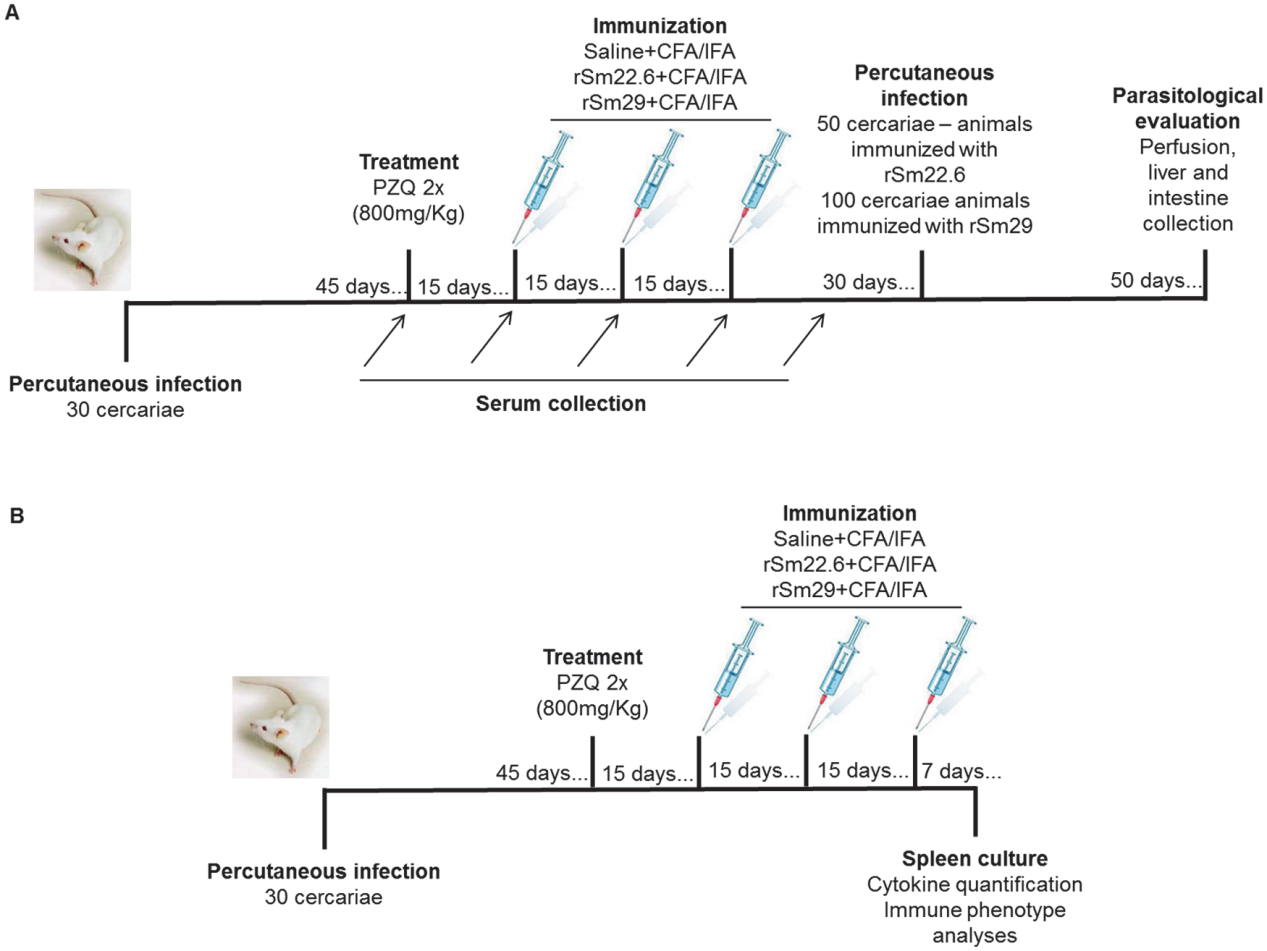
Experimental design. The assessment of the protection (A) and the immune response (B) triggered by rSm22.6 or rSm29 immunization in previously infected and treated Balb/c mice were performed as indicated in the figure.

Mice were infected through percutaneous exposure of shaved abdominal skin for 1h in water containing approximately 30 cercariae, as previously described [16]. After forty-five days, mice were treated with two oral doses of 800mg/Kg Praziquantel, with an interval of five days between doses to ensure that all parasites were killed. Fifteen days after treatment, mice were separated into four groups (IT/rSm22.6; IT/Saline(rSm22.6); IT/rSm29; IT/Saline(rSm29) of ten animals each. Additionally Balb/c naïve mice were used in immunization protocols to evaluate the protection triggered by rSm22.6 and rSm29 in this mouse strain. Immunization protocol consisted in three doses of the vaccine injected subcutaneously in the nape of the neck with rSm22.6 (25μg/animal); rSm29 (25μg/animal) or saline plus Freund’s adjuvant. Mice received immunization doses in a fifteen-day interval regimen. In the first dose, mice were immunized with Complete Freund’s Adjuvant (CFA) and in the subsequent boosters, Incomplete Freund’s Adjuvant (IFA) was used. Thirty days after the last booster, animals were challenged through percutaneous infection with 100 cercariae (IT/rSm29; IT/Saline(rSm29); rSm29; Saline(rSm29) groups) or 50 cercariae (IT/rSm22.6; IT/Saline(rSm22.6); rSm22.6; Saline(rSm22.6) groups). The number of cercariae used in challenge infections was similar to the number of those used in pre-clinical trials using rSm22.6 or rSm29 immunization in C57BL/6 naïve mice [9,12] (Fig. 1).

### Worm burden recovery

Fifty days after challenge infection, adult worms were perfused by portal veins as described by Pellegrino and Siqueira [17]. Protection level was calculated comparing the number of worms recovered from the immunized group (IT/rSm22.6, IT/rSm29, rSm22.6 or rSm29) with the number of worms recovered in the saline control groups (IT/Saline(rSm29), IT/Saline(rSm22.6), Saline(rSm22.6) or Saline(rSm29)), using the formula below:

Level Protection:

$$\frac{\text{Burden recovered from control group - Burden recovered from experimental group}}{\text{Burden recovered from control group}}\times 100$$

### Egg count and histophatological analysis

Following perfusion, the intestine from infected, treated and vaccinated animals (IT/rSm22.6 or IT/rSm29) and their control group (inoculated with saline) were removed, weighed and digested with 10% KOH overnight at room temperature. The eggs were obtained by centrifugation at 900 x g for 10 min and were resuspended in 1 mL of saline. The number of eggs present in intestine was determined using a light microscope

Liver sections from IT/rSm29 immunized mice and from its control group (IT/Saline) were collected following perfusion. The liver sections removed from the central part of the lateral lobe were fixed in 10% buffered formaldehyde in phosphate buffered saline (PBS). Histological sections were stained with Haematoxylin and Eosin (HE). To determine granuloma area, approximately 100 granulomas from each group (10 granulomas/animal) with a single well-defined egg and at exudative stage were randomly chosen at 10x objective lens through an AxioCam microcamera (Carl Zeiss, Germany). Using the AxionVision 4.8 image analysis software (Carl Zeiss MicroImaging GmbH, Germany), the total area of the granulomas were measured, and the results were expressed in square micrometers (μm^2^).

### Measurement of specific antibodies

Serum from mice of each of the different groups were obtained forty five days after infection, fifteen days after treatment and fifteen days after each immunization dose in individual basis. Sera were used to determine the production of IgG, IgG1, IgG2a and IgE specific antibodies by ELISA. Briefly, MaxiSorp 96-well microtiter plates (Nunc) were coated with rSm22.6 or rSm29 at a concentration of 5μg/mL (IgG, IgG1 and IgG2a), or 1 μg/mL (IgE) in carbonate-bicarbonate buffer, pH 9.6, over night at 4°C. Then, the plates were blocked with 300μL/well phosphate-buffered saline, pH 7.2, with 0.05% Tween-20 (PBST) plus 3% FBS (fetal bovine serum, GIBCO, USA), for 2 hours at room temperature (IgG, IgG1 and IgG2a), or with PBST plus 3% nonfat dry milk over night at 4°C (IgE). One hundred microliters/well of pool of sera samples from each group was submitted to serial dilution beginning at 1:20 and ending in 1:1.310.720, in duplicate, to determine antibody titers and to standardize the dilution of sera used in individual basis. Analyzes of antibody levels in individual basis were performed using one hundred microliters/well of each serum sample, diluted 1:1.000 (IgG and IgG1—rSm29 tests), 1:100 (IgG2a—rSm29 test), 1:600 (IgG—rSm22.6 test), 1: 1.000 (IgG1—rSm22.6 test), 1:400 (IgG2a—rSm22.6 trial) or 1:40 (IgE—rSm22.6 and rSm29 test). Finally, the plates were incubated with peroxidase-conjugated anti-mouse IgG, IgG1 and IgG2a (Southern Biotech, USA), diluted 1:10.000, 1:10.000 and 1:8.000, respectively, for 1h at room temperature (RT). For IgE measurement, an anti-mouse biotin IgE (BD Pharmingen) diluted 1:250 was used and, after incubation (1h at RT), an avidin conjugated to peroxidase (1:250) was added for 30 minutes. Color reaction was developed by TMB incubation (Microwell Peroxidase Substrate System) and stopped with 5% sulfuric acid. The plates were read at 450 nm in an ELISA plate reader (Bio-Rad, USA).

### Cytokine quantification and immune phenotype analyses

Spleens from IT/Saline, IT/rSm29 or IT/rSm22.6 immunized animals were obtained 7 days after the last immunization dose (Fig. 1). As a control for the experiment, spleens from naïve Balb/c mice immunized with rSm22.6 or rSm29 or from non-immunized infected and treated mice were also obtained. Red cells were lysed with ACK, and spleen cells were washed twice with apyrogenic saline and adjusted to 1x10^6^ cells/well. Cells were cultured in 5% CO_2_ at 37°C in the presence of medium, Concanavalin (5μg/mL), rSm29 or rSm22.6 (25μg/mL). Culture supernatants were collected 24 or 72 hours post stimulation for IL-4 (24h), IL-6 (24h), IL-10 (72h), IL-17 (72h), IFN-γ (72h) and TNF-α (24h) evaluation. Cytokine measurement was performed using Cytometic Bead Array Kit, anti-mouse CBA Th1/Th2/Th17 Kit (BD Pharmingen, USA). CBA was performed according to the manufacturer’s protocol. Beads were acquired in FACScalibur flow cytometer (BD, USA) and data were analyzed using FCAP Array Software (Becton Dickinson). For *ex vivo* analyses, spleen cells were adjusted to 5x10^6^ cells/well and incubated for 15 min at 4°C with anti-mouse CD16/CD32 mAbs (Fc-Block—BD-Pharmingen) to block Fc gamma receptors. After a wash step with PBS (Sigma), cells were incubated for 15 min at 4°C with antibodies against surface molecules: anti-CD4 (BD-Pharmingen, clone GK1.5), anti-CD8 (BD-Pharmingen, clone 53–6.7), anti-F4/80 (eBioscience, clone BM8) and anti-IgG (eBioscience, clone eBio299Arm) conjugated to FITC; anti-CD69 (eBioscience, clone H1.2F3), anti-CD86 (Accurate Chemical and Scientific Corporation, Westbury, NY, USA) and anti-CD4 (BD-Pharmingen, clone GK1.5) conjugated to PE; anti-CD27 (eBioscience, clone LG.7F9) and anti-CD25 (BD-Pharmingen, clone 7D4) conjugated to biotin; anti-CD19 (BD-Pharmingen, clone 1D3) and anti-CD127 (BD-Pharmingen, clone A7R34) conjugated to PECy7; anti-CD44 (BioLegend, clone IM7) conjugated to Pacific Blue and anti-CD62L (BD-Pharmingen, clone MEL-14) conjugated to Alexa 700. After that, cells were washed with PBS (0.15M), BSA (0.5%) and NaN_3_ (2mM) and incubated for 20 min with streptavidin-APC (1:200) at 4°C. Subsequently, cells were washed and fixed using 2% formaldehyde solution and were acquired using LSRFortessa (Becton Dickinson, San Jose, CA). Data analysis was performed using FlowJO software (TreeStar, Ashland).

### Recognition of Sm22.6 and Sm29 on parasite surface by sera from immunized mice

To assess the expression of Sm22.6 and Sm29 on parasite surface, skin-stage schistosomula were incubated with sera from Balb/c naïve mice immunized with three doses of rSm22.6 or rSm29 in Freunds’ adjuvant. Cercariae were mechanically transformed into skin-stage schistosomula as previously described [18], with some modifications. Briefly, cercariae were incubated on ice for 30 min, centrifuged (1800g/3 min/4°C), and suspended in cold Glasgow medium (Sigma-Aldrich, St. Louis, MO, USA) plus 1% penicillin/streptomycin and 10% FBS. Cercariae tails were sheared off by vortexing for 2 min in high speed and were removed through several washing steps with Glasgow medium. The schistosomula were cultured for 3 hours at 37°C in medium. Later, the schistosomula were incubated with immune sera and FITC-conjugated anti-mouse IgG, as described [19]. Briefly, schistosomula were washed three times with DMEM medium by centrifugation at 1800g/5 min. Subsequently, the parasites were fixed with PBS plus 1% formaldehyde for 1 hour at 4°C. After this, schistosomula were washed three times with PBS and incubated in RPMI for 30 min, and in PBS + 1% BSA for additional 30 min. After another washing step, schistosomula were incubated with sera from Sm22.6 and Sm29 immunized mice diluted 1:50 in PBS overnight at 4°C. Afterwards, schistosomula were washed with PBS + 1% BSA (3x), incubated in RPMI for 30 min, washed with PBS (3x) and incubated in PBS + 1% BSA for 30 min. Finally, schistosomula were incubated with an anti-IgG FITC-conjugated antibody for 2 hours in the dark, and antibodies binding to schistosomula surface were evaluated by fluorescence microscopy. The fluorescence intensity in the schistosomula tegument was measure using ImageJ software and the fluorescence analysis was determinate as described before [20].

### Statistical analysis

Data normality was tested using the D’Agostino-Pearson omnibus test. Statistical analyses were performed using the Mann-Whitney nonparametric test for cytokine measurement and *ex vivo* analyses or Student’s t-test for parasitological analyses and antibodies measurement using the software package GraphPad Prism 5.0 (Graph-Pad Software, San Diego, CA, USA).

## Results

### Immunization with rSm29 triggers protection in Balb/c infected/treated animals

**Fig 2:**
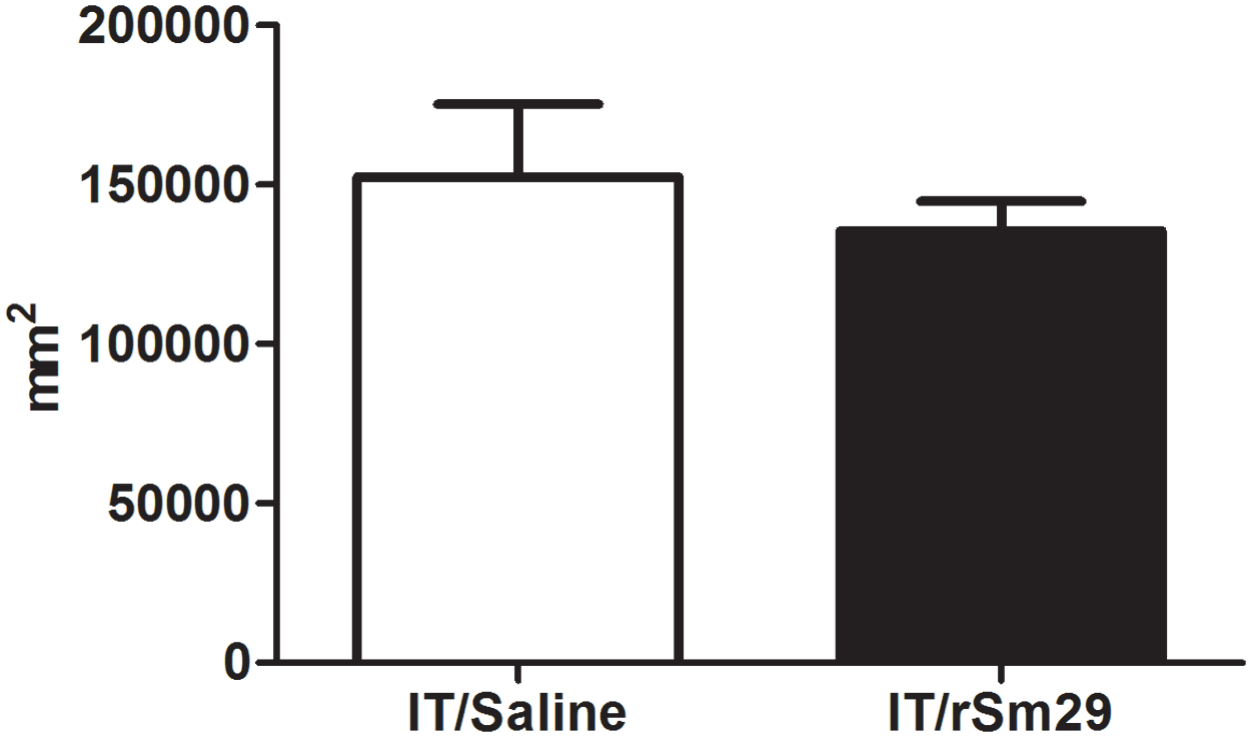
Hepatic granuloma area in mice immunized with rSm29, previous infected and treated. Representative picture of granuloma reaction (A) and the measurement of granuloma area of each group (B). To determine granuloma area, approximately 100 granulomas from IT/rSm29 group and from its control group (IT/Saline), with a single well-defined egg (exudative stage), were measured. Total area of the granulomas was expressed in square micrometers (μm^2^). Scale bar = 100 μm (x100).

The level of protection induced by rSm22.6 or rSm29 immunization in Balb/c mice, previously sensitized by *S*. *mansoni* infection and Praziquantel treatment, was evaluated 50 days after challenge infection with 50 (IT/rSm22.6 trials) or 100 (IT/rSm29 trials) *S*. *mansoni* cercariae. In order to establish the number of immunization doses required to induce a protective immunity, parasite burden after challenge infection was evaluated in mice immunized with one, two or three doses of vaccine formulations. No significant reduction in worm burden was observed in mice immunized with rSm22.6 (IT/rSm22.6) compared to the control group regardless of the number of vaccination doses received (Table 1). In addition, IT/rSm22.6 group also showed no significant reduction in eggs trapped in intestine (Table 1). On the other hand, mice immunization with 3 doses of rSm29 (IT/rSm29 group) induced a significant reduction (26–48%) in worm burden in comparison to saline control group (Table 2). A significant decrease in the numbers of eggs recovered from the intestine of the IT/rSm29 immunized group was observed in comparison with saline group (Table 2), reflecting the reduction in worm burden in this group. Despite this, the immunization with rSm29 in previously infected and treated mice failure to reduce granuloma area (Fig. 2). Interestingly, in naïve Balb/c mice neither immunization with rSm22.6 nor immunization with rSm29 induced reduction in parasite burden (Table 3).

**Table 1:** Protection level induced by immunization with rSm22.6 plus Freund’s adjuvant in Balb/c mice previously infected/treated.

	Worm burden recovery				Egg/gram of Intestine
	Male Mean±SD	Female Mean±SD	Total Mean±SD	Protection (%) ^b^	Mean±SD ^c^
**Trial 1** ^**a**^					
IT/Saline (1 dose)	8 ± 5	7 ± 4	15 ± 7		ND
IT/rSm22.6 (1 dose)	7 ± 2	6 ± 2	13 ± 3	13% (NS)	ND
IT/Saline (2 doses)	11 ± 7	7 ± 5	18 ± 11		ND
IT/rSm22.6 (2 doses)	7 ± 4	6 ± 3	14 ± 6	22% (NS)	ND
IT/Saline (3 doses)	11 ± 4	9 ± 5	21 ± 5		10960±6546
IT/rSm22.6 (3 doses)	10 ± 3	8 ± 5	18 ± 8	15% (NS)	7713±6538 (NS)
**Trial 2** ^**a**^					
IT/Saline (1 dose)	10 ± 6	10 ± 5	20 ± 11		ND
IT/rSm22.6 (1 dose)	10 ± 6	8 ± 4	17 ± 10	15% (NS)	ND
IT/Saline (2 doses)	8 ± 5	8 ± 4	15 ± 9		ND
IT/rSm22.6 (2 doses)	8 ± 3	8 ± 3	16 ± 6	0% (NS)	ND
IT/Saline (3 doses)	10 ± 4	13 ± 6	23 ± 9		8579±5853
IT/rSm22.6 (3 doses)	11 ± 6	11 ± 6	27 ± 7	0% (NS)	8433±5880 (NS)

^a^Worm burden recovered from mice immunization with one, two or three doses. For each vaccinated group: n = 10 mice.

Comparison between total worm burden recovered^b^ or numbers of eggs per gram of intestine^c^ from IT/Sm22.6 group and IT/Saline control group, which received the same number of immunization doses and were challenged with 50 *S*. *mansoni* LE strain cercariae. NS = not significant. ND = not determined.

**Table 2:** Protection level induced by immunization with rSm29 plus Freund’s adjuvant in Balb/c mice previously infected/treated animals.

	Worm Burden recovery				Egg/gram of Intestine
	Male Mean±SD	Female Mean±SD	Total Mean±SD	Protection(%) ^b^	Mean±SD ^c^
**Trial 1** ^**a**^					
IT/Saline(1 dose)	24 ± 8	24 ± 6	43 ± 17		ND
IT/rSm29(1 dose)	19 ± 8	20 ± 8	39 ± 16	9%(NS)	ND
IT/Saline(2 doses)	22 ± 4	21 ± 3	43 ± 7		ND
IT/rSm29(2 doses)	20 ± 5	18 ± 4	38 ± 8	12%(NS)	ND
IT/Saline(3 doses)	25 ± 7	26 ± 8	51 ± 15		23660±3602
IT/rSm29(3 doses)	15 ± 5	17 ± 6	35 ± 7	31%*(p = 0.03)	14780±5463*(p = 0.0021)
**Trial 2** ^**a**^					
IT/Saline(3 doses)	20 ± 7	20 ± 8	40 ± 14		18040±11080
IT/rSm29(3 doses)	11 ± 7	10 ± 6	21 ± 13	48%*(p = 0.01)	6061±4145*(p = 0.0125)
**Trial 3** ^**a**^					
IT/Saline(3 dose)	20 ± 4	22 ± 4	42 ± 8		ND
IT/rSm29(3 dose)	15 ± 4	16 ± 4	31 ± 7	26%*(p = 0.02)	ND

^a^Worm burden recovered from mice immunization with one, two or three doses. For each vaccinated group: n = 10 mice.

Comparison between total worm burden recovered^b^ or numbers of eggs per gram of intestine^c^ from IT/rSm29 group and IT/Saline control group, which received the same number of immunization doses and were challenged with 100 *S*. *mansoni* LE strain cercariae. NS = not significant. ND = not determined.

*Statistically significant (p<0.05)

**Table 3:** Protection level induced by immunization with rSm29 or rSm22.6 plus Freund’s adjuvant in Balb/c naïve mice.

	Worm Burden recovery			
	Male Mean±SD	Female Mean±SD	Total Mean±SD	Protection (%) ^c^
**Sm22.6 experiment** ^**a**^				
**Trial 1**				
Saline (3 doses)	10 ± 6	7 ± 4	17 ± 9	
rSm22.6 (3 doses)	12 ± 7	9 ± 5	17 ± 10	0% (NS)
**Trial 2**				
Saline (3 doses)	14 ± 5	13 ± 5	27 ± 10	
rSm22.6 (3 doses)	11 ± 6	10 ± 6	22 ± 10	18% (NS)
**Sm29 experiment** ^**b**^				
**Trial 1**				
Saline (3 doses)	17 ± 7	15 ± 7	32 ± 14	
rSm29 (3 doses)	18 ± 7	14 ± 6	32 ± 13	0% (NS)
**Trial 2**				
Saline (3 doses)	23 ± 5	22 ± 7	45 ± 12	
rSm29 (3 doses)	26 ± 9	21 ± 5	46 ± 11	0% (NS)

^a^Worm burden recovered from mice infected with 50 cercariae.

^b^Worm burden recovered from mice infected with 100 cercariae. For each vaccinated group: n = 10 mice.

^c^Comparison between total worm burden recovered from rSm29 or rSm22.6 group and Saline control group.

### Immunization with rSm22.6 and Sm29 induce a vigorous humoral response

**Fig 3:**
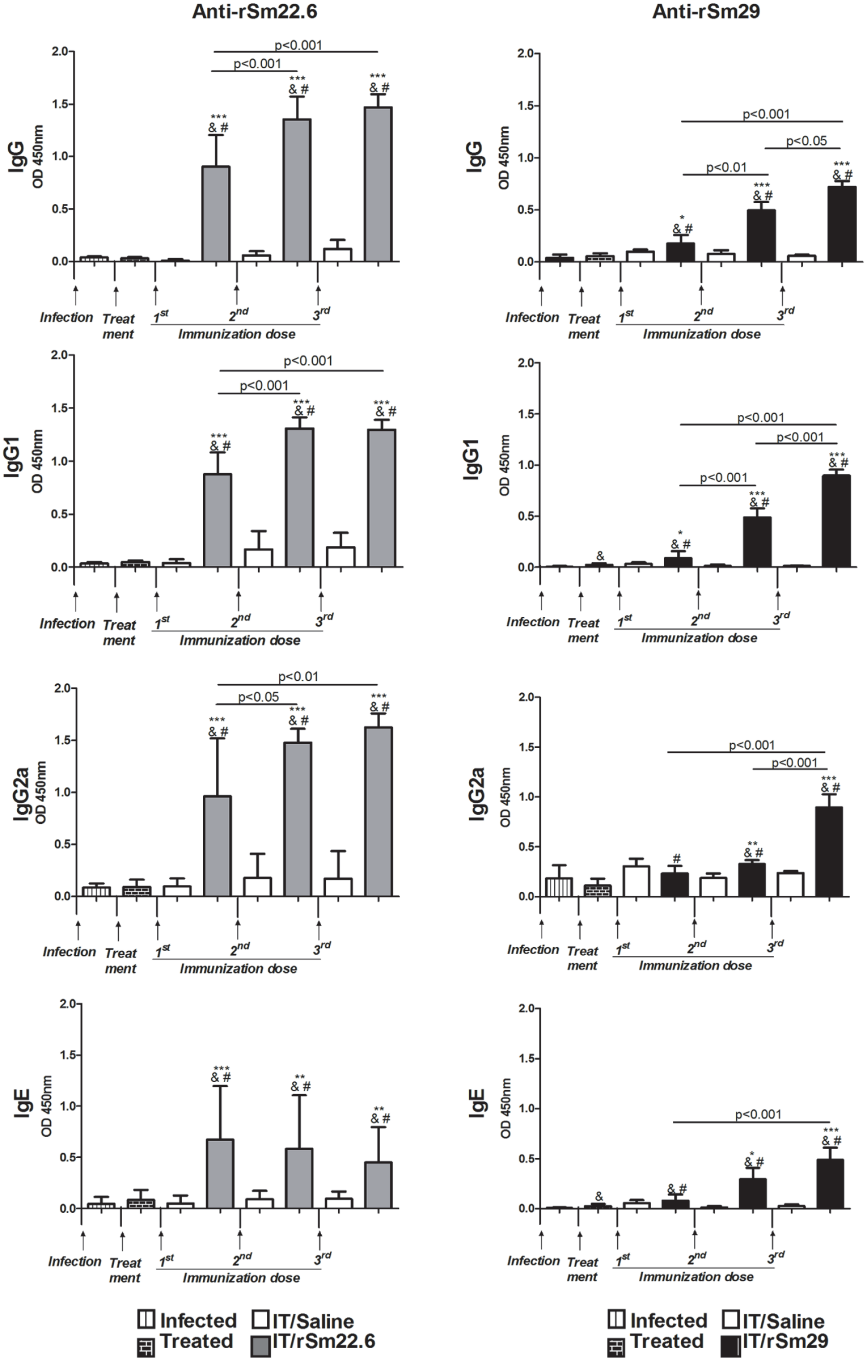
Kinetics of specific anti-rSm22.6 or anti-rSm29 antibodies production induced by immunization. Sera from 10 animals/group were collected forty-five days after infection, fifteen days after treatment and two weeks after each immunization dose. The levels of specific IgG, IgG1, IgG2a, IgE against rSm22.6 or against rSm29 were determined by ELISA. Sera dilution was: 1:1.000 (IgG and IgG1—rSm29 tests), 1:100 (IgG2a—rSm29 test), 1:600 (IgG—rSm22.6 test), 1: 1.000 (IgG1—rSm22.6 test), 1:400 (IgG2a—rSm22.6 test) or 1:40 (IgE—rSm22.6 and rSm29 test). Bars represent the mean absorbance values measured at 450 nm ± SD (Standard Deviation). Arrows indicate the timing of infection, treatment and immunization. Statistically significant differences between rSm22.6 or rSm29 group and saline control group is denoted in the graphic by one asterisk (p<0.05), two asterisks (p<0.01) or three asterisks (p<0.001). Statistically significant difference between the immunization doses is pointed in the graphic. &: difference compared to infected mice; #: difference compared to treated mice.

A significantly higher production of specific anti-rSm22.6 IgG, IgG1, IgG2a and IgE was observed in IT/rSm22.6 immunized mice compared to control group (IT/Saline) in all of the time points evaluated and also compared to infected and infected/treated mice (Fig. 3). Furthermore, in the IT/rSm22.6 immunized group, the levels of specific IgG, IgG1 and IgG2a were significantly increased by the second and the third doses of the vaccine (Fig. 3). In IT/rSm29 immunized mice, significantly higher production of specific IgG and IgG1 in comparison to control group was observed in all of the time points evaluated (Fig. 3), while a significantly higher production of specific IgG2a and IgE in comparison with control group was only detected after the second immunization dose (Fig. 3). Moreover, in IT/rSm29 immunized group, a significant increase in IgG and IgG1 production was observed after each booster (Fig. 3), while a significant increase in specific anti-rSm29 IgG2a levels was observed after the third immunization dose in comparison with the first and the second doses of the vaccine (Fig. 3). The levels of anti-rSm29 IgE, in turn, increased only after the third immunization dose, compared to the first dose (Fig. 3). Also, a significant increase in anti-rSm29 IgG1 and IgE levels was observed in infected/treated mice compared to infected animals. Immunization with rSm29 significantly increased the levels of IgG, IgG1, IgG2a and IgE in comparison with the levels detected in infected or infected/treated mice.

The titers of specific Sm22.6 IgG did not differ between naïve immunized mice and infected/treated immunized mice (Table 4). But the titer of specific Sm22.6 IgG2a and IgG1 antibodies was lower in mice immunized after a previous infection whereas IgE titer was higher in these animals (Table 4). A higher titer of Sm22.6-specific IgG, IgG1, IgG2a and IgE antibodies was observed in infected/treated immunized mice compared to infected/treated Saline inoculated animals (Table 4). In rSm29 immunized mice, the titers of Sm29-specific IgG2a did not differ between naïve immunized mice and infected/treated immunized mice (Table 4). A lower titer of specific IgE and IgG1 was observed in mice that had been previously infected and treated, while higher IgG titers were observed in these animals in comparison with the naïve immunized mice (Table 4). As in rSm22.6 immunized mice, a higher titer of IgG, IgG1, IgG2a and IgE antibodies was observed in infected/treated immunized group compared to infected/treated Saline group (Table 4).

**Table 4:** Antibody titer in mice immunized with rSm22.6 and rSm29 after the third immunization.

	rSm22.6	IT/Saline	IT/rSm22.6	Fold increase IT/rSm22.6/ IT/Saline	rSm29	IT/Saline	IT/rSm29	Fold increase IT/rSm29/IT/Saline
IgG	1: 1.310.720	1: 10.240	1: 1.310.720	128X	1: 163.840	1: 10.240	1: 655.360	64X
IgG1	1: 2.621.440	1: 10.240	1: 1.310.720	128X	1: 1.310.720	1: 5.120	1: 655.360	128X
IgG2a	1: 653.360	1: 2.560	1: 163.840	64X	1: 40.960	1: 2.560	1: 40.960	16X
IgE	1: 1.280	1: 320	1: 5.120	16X	1: >1.280	1: 20	1: 320	16X

### Antibodies induced by recombinant Sm22.6 and Sm29 recognized native proteins on parasite surface

**Fig 4:**
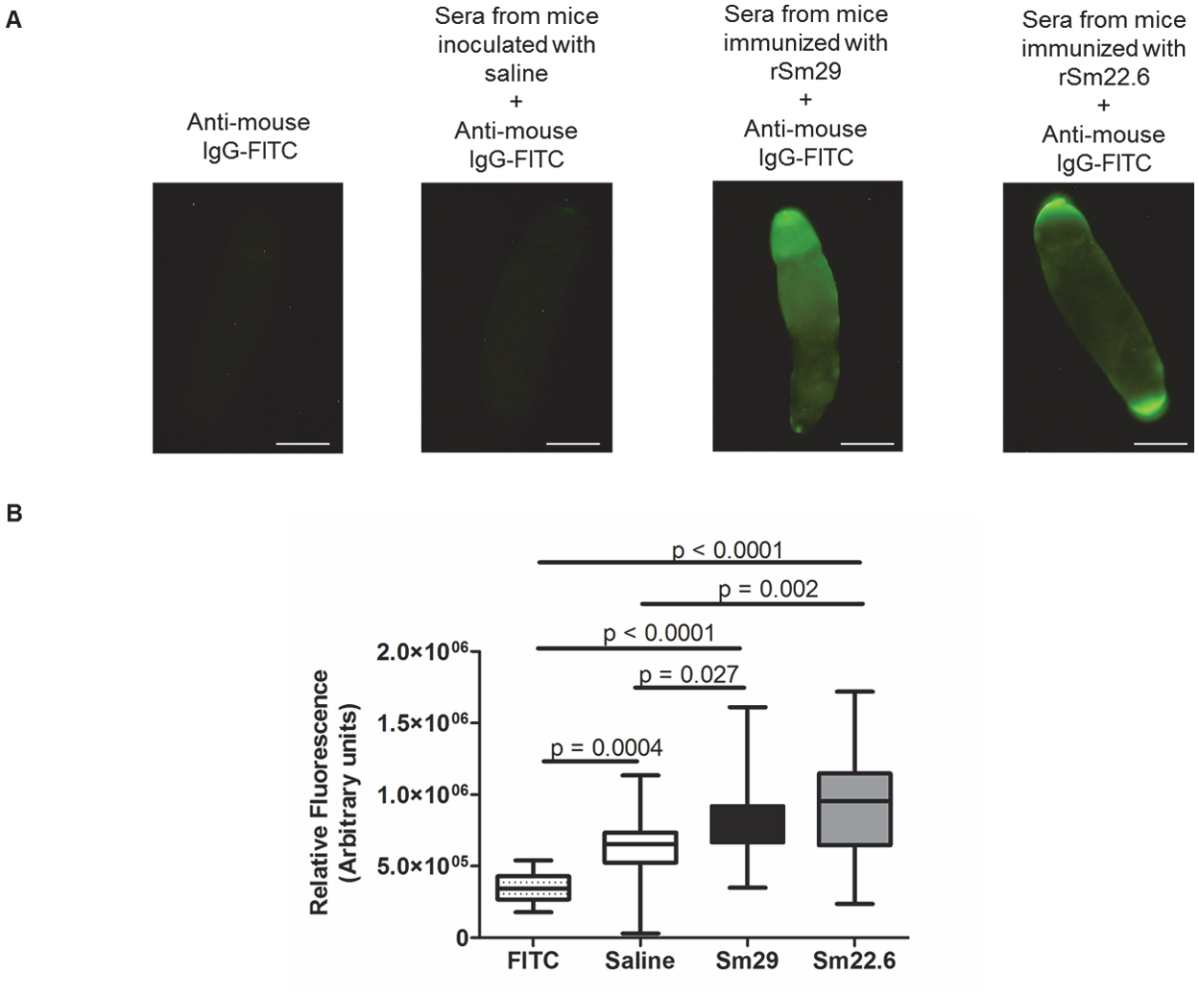
Recognition of native Sm22.6 and Sm29 in schistosomula surface by sera from immunized mice. Twenty newly-transformed schistosomula (A) were incubated with sera from mice inoculated with saline + CFA/IFA, immunized with rSm29 + CFA/IFA or immunized with rSm22.6 + CFA/IFA. Antibody reactivity to Sm29 and Sm22.6 surface proteins were detected by a secondary anti-mouse IgG-FITC-conjugated antibody. As an experimental control, parasites were incubated with anti-mouse IgG-FITC-conjugated antibody. The fluorescence in the schistosomula tegument, after incubation, was observed by fluorescence microscopy. Scale bar = 25 μm (x400). A significant fluorescence measurement was detected (B) in the schistosomulum incubated with sera from rSm22.6 and rSm29 immunized mice compared to parasite incubated with sera from saline inoculated animals. Fluorescence intensity in the schistosomula tegument was measure using ImageJ software. The graphic represent box plot with whiskers from minimum to maximum values of relative fluorescence (Arbitrary unit). Significant differences were denoted in the graphic.

To evaluate whether the antibodies produced in response to mice immunization with the recombinant form of Sm22.6 and Sm29 were able to recognize the native proteins in the parasite surface, an immunofluorescence staining assay was performed in newly-transformed schistosomula. Fig. 4A shows a representative picture of the results observed in immunofluorescence staining and demonstrates that the antibodies raised against the recombinant form of Sm22.6 or Sm29 recognize the native protein expressed on the parasite surface, since significant fluorescence measurement is detected in the presence of sera from rSm22.6 and rSm29 immunized mice compared to sera from saline inoculated animals (Fig. 4B). Nonspecific recognition was not observed on parasites incubated with FITC conjugated anti-mouse IgG antibody (Fig. 4B).

### Immunization with rSm22.6 and rSm29 induced a mixed type of immune response

**Fig 5:**
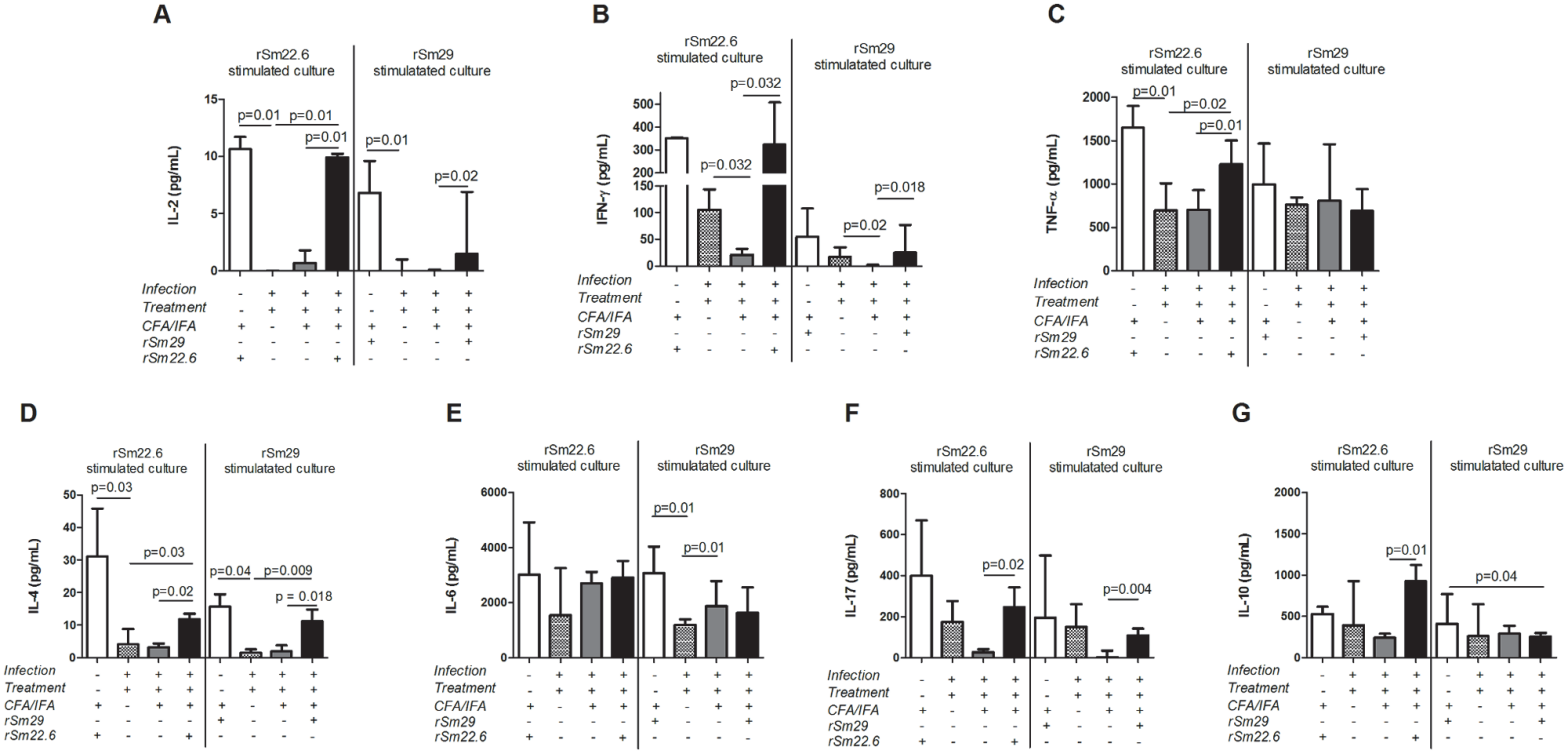
Cytokine profile induced by immunized Balb/c mice, previously infected/treated, with the recombinant form of Sm22.6 and Sm29. One week after the last immunization dose, spleen cells were obtained and cultured for cytokine measurement. Spleens from naïve mice immunized with rSm22.6 or rSm29 (white bars) or from non-immunized infected and treated mice (cross-hatched bars) were also obtained as a control for the experiment. IL-2 (A), TNF-α (B), IFN-ɣ (C), IL-4 (D), IL-6 (E), IL-17 (F) and IL-10 (G) production in response to rSm22.6 (25μg/mL), rSm29 (25μg/mL) or medium alone (control) was assessed. Cytokine levels were measured by the CBA Th1/Th2/Th17 Kit. Bars represent the median with interquartile range of the difference on cytokine production in response to rSm22.6 or rSm29 and the basal cytokine production (observed in nonstimulated splenocytes); gray bars represent the cytokine production in the saline group in response to rSm22.6 or rSm29 in vitro stimulation; black bars represent the cytokine production in rSm29 or rSm22.6 immunized group in response to rSm22.6 or rSm29 in vitro stimulation. Statistically significant difference between saline and rSm22.6 or Sm29 group is pointed in the graphic. Statistical analyses were performed using Mann-Whitney test.

To determine the cytokine profile induced by immunization, the levels of IL-2, IFN-γ, TNF-α, IL-4, IL-6, IL-10 and IL-17 in the supernatant of spleen cells culture wer measured. In the group of mice immunized with three doses of rSm22.6 (IT/Sm22.6), rSm22.6 *in vitro* stimulation induced a significant production of IL-2, IFN-γ, TNF-α, IL-4, IL-10 and IL-17 in comparison to saline group (Fig. 5). Differences in the production of IL-2, TNF-α and IL-4 were also observed between non-immunized mice that had been infected with *S*. *mansoni* and treated with Praziquantel and Sm22.6 immunized mice, with the higher production of those cytokines being observed in the immunized groups (Fig. 5). In the animals immunized with three doses of rSm29 (IT/Sm29) *in vitro* re-stimulation induced significantly higher production of IL-2, IFN- γ, IL-17 and IL-4 in comparison to saline control group and significantly higher IL-4 production in comparison to non immunized infected/treated animals (Fig. 5). The cytokine profile induced by rSm22.6 or rSm29 immunization in mice previously exposed to parasite antigen was similar to the one observed in naïve immunized mice, except for the IL-10 production, which was reduced in rSm29 immunized mice that had been previously infected and treated (Fig. 5).

### Mice immunized by rSm29 induced higher percentage of memory cells

**Fig 6:**
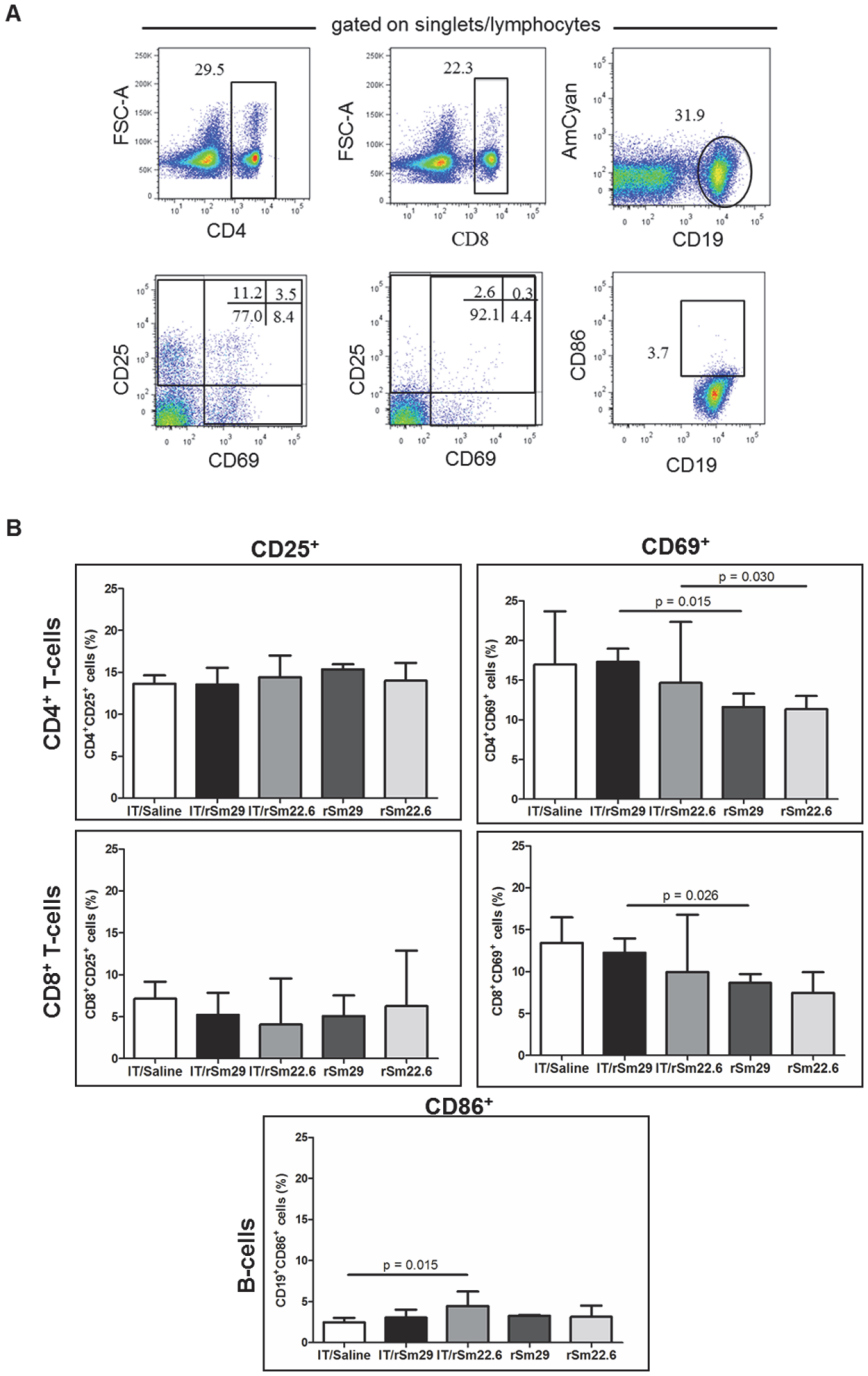
Activation status of spleen cells from mice immunized with rSm29 or rSm22.6, or inoculated with saline. One week after the last immunization, spleen cells from IT/Saline, IT/rSm22.6, IT/rSm29, rSm22.6 and rSm29 groups were labeled to evaluate the frequency of activated lymphocytes, and were acquired in a flow cytometer. Data analysis was carried out as follows (A): within singlet cells/lymphocyte region, cells expressing CD4, CD8 or CD19 molecules were selected and the expression of CD25 and CD69 activation molecules were evaluated in TCD4^+^ or TCD8^+^ cells. B-cells activation status was evaluated in CD19+ cells expressing the activation marker CD86. (B): Bars represent the median with interquartile range of the percentage of cells expressing the activation molecules. Statistically significant difference is pointed in the graphic. Statistical analyses were performed by the Mann-Whitney test.

**Fig 7:**
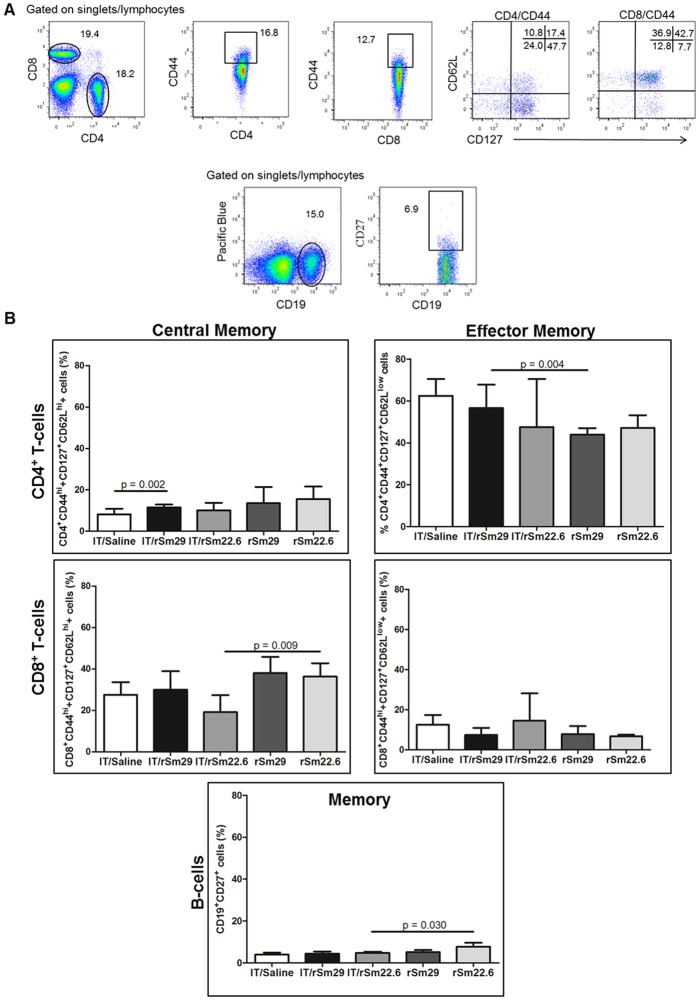
Frequency of memory cells in mice immunized with rSm29 or rSm22.6, or inoculated with saline. One week after the last immunization, spleen cells from IT/Saline, IT/rSm22.6, IT/rSm29, rSm22.6 and rSm29 groups were labeled to assess the frequency of memory lymphocytes and were acquired in flow cytometer. Data analysis was carried out as follows (A): within singlet cells/lymphocyte population, CD4+CD44^high^ or CD8CD44^high^ cells were selected and, within that population, the percentage of CD127^+^CD62^low^ cells representing CD4^+^ or CD8^+^ T effector memory cells, CD127^+^CD62^high^ population representing the CD4^+^ or CD8^+^ T central memory cells were determined. Furthermore, within the lymphocytes population, CD19^+^ cells were selected and the percentage of CD19^+^CD27^+^ cells was determined. (B): The results are expressed in bars as median with interquartile range of the percentage of memory cells. Statistically significant difference is pointed in the graphic. Statistical analyses were performed by the Mann-Whitney test.

The expression of CD25, CD69 and CD86 activation markers was evaluated on the surface of lymphocytes. A higher percentage of activated B cells (CD19^+^CD86^+^) was observed in IT/Sm22.6 mice in comparison with IT/Saline group, while no difference was observed in TCD4^+^ and TCD8^+^ activation between IT/Saline group and IT/rSm22.6 or IT/rSm29 (Fig. 6). When compared to naïve Balb/c mice, immunization of infected/treated animals with rSm29 increase significantly the percentage of CD4+CD69+ and CD8+CD69+ cells, while immunization with rSm22.6 increase significantly the percentage of CD4+CD69+ cells (Fig. 6). Regarding memory cells, no differences in the percentage of CD8^+^ central memory cells (CD44^hi^CD62^hi^ CD127^+^), T CD4^+^ or CD8^+^ effector memory cells (CD44^hi^CD62^lo^CD127^+^) and memory B cells (CD19^+^CD27^+^) was observed between IT/rSm29 or IT/rSm22.6 and IT/Saline group (Fig. 7). A higher percentage of central memory TCD4+ lymphocytes were observed in the IT/rSm29 immunized group compared to control group (Fig. 7). Compared to naïve Balb/c mice, immunization with rSm29 in infected and treated animals induced significant higher percentage of CD4+ effector memory cells (Fig. 7). In contrast naïve Balb/c mice immunized with rSm22.6 presented a higher percentage of CD8+ central memory and memory B cells then IT/rSm22.6 group (Fig. 7). Furthermore, regarding monocytes activation status (F4/80^+^CD86^+^), no difference between immunized groups (rSm22.6 or rSm29) and control group was observed (S1 Fig.).

## Discussion

Currently, many efforts are focused on the schistosomiasis vaccine development, which will effectively contribute to disease control and eradication. Many vaccine candidates have been indentified and tested in pre-clinical trials with promising results. For instance, the recombinant proteins Sm22.6 or Sm29 induced significant worm burden reduction in C57BL/6 immunized mice [9,12,21].

In the case of schistosomiasis, the population most affected by the disease, and thus, the target to an anti-schistosomiasis vaccine, is represented by the residents of endemic areas, who have persistent contact with the parasite through constant infections/treatments throughout life responding differently to antigen stimulation if compared to an individual not sensitized previously by parasite antigens. Therefore it would be interesting, before moving forward to clinical trials, to evaluate promising vaccine candidates using experimental models that have already had previous contact with the parasite, approximating the vaccine research to the endemic area reality.

Herein we performed an immunization protocol using Balb/c mice previously infected with 30 *S*. *mansoni* cercariae and treated with Praziquantel. Balb/c strain was the strain of choice in our studies since its Th2 genetic background resembles the immunological profile identified in individuals living in endemic areas for schistosomiasis [13,14,15,22]. Our results demonstrate that three doses of the vaccine containing rSm29 were necessary to elicit significant protection in mice previously exposed to parasite antigens, reducing the number of the worms recovered. This reduction reflected in significant decrease in the numbers of eggs recovered from the intestine. Although a reduction in parasite burden was observed in our study, immunization had no effect in liver pathology, thus this vaccine formulation would have an impact on disease transmission rather than in individual pathology. Immunization with Sm22.6, on the other hand, failed to induce protection even after three doses of the vaccine. Since immunization of naïve Balb/c mice with rSm29 or rSm22.6 has never been evaluated, we also assessed the ability of both antigens to induce protection in this mice strain, interestingly neither rSm22.6 nor rSm29 was able to induce significant reduction in worm burden.

Some studies have demonstrated the importance of antibodies in the protection induced by immunization. These studies demonstrate that antibodies are involved in parasite elimination [19,23,24]. Our results show a vigorous humoral immune response, with significant levels of IgG, IgG1 and IgG2a, and IgE in infected/treated Balb/c mice immunized with rSm22.6 or rSm29 plus Freund’s adjuvant. The humoral response induced by rSm22.6 immunization was observed after the first dose received. Our results corroborate with previous studies using rSm22.6 in vaccine formulations with Freund’s or Alum in naïve C57BL/6 mice, which showed a significant IgG production from the first immunization dose [9,21]. However, as in mice immunized with rSm22.6 + Alum [21], we did not observe a significant reduction in parasite burden, suggesting that the IgG immunoglobulin induced by rSm22.6 immunization did not play a key role in worm elimination in these animals.

Many studies have demonstrated the importance of immunoglobulin IgE in the parasite elimination and in the resistance to reinfection in individuals living in endemic areas [7,25,26,2,15,6]. An important mechanism involved in parasite elimination is mediated by IgE and eosinophils: in the presence of IgE, eosinophils can kill *S*. *mansoni* larvae through the antibody-dependent cytotoxicity (ADCC) process [25,27]. In this context, some studies demonstrate that individuals who present greater resistance to reinfection exhibit elevated IgE levels, associated with low levels of IgG4 against parasite antigens [2,6]. Indeed, many of these studies demonstrated that this resistance is mainly related to the response against the Sm22.6 (SmTAL1) protein [28,15,29,30,31,32]. These studies revealed that, after treatment, individuals presented higher anti-Sm22.6 IgE levels, probably due to increased Sm22.6 exposure to the host immune system after parasite death. Indeed a higher titer of Sm22.6 specific IgE antibody was observed in infected and treated Sm22.6 immunized animals compared to rSm22.6 naïve immunized mice. However vaccine booster did not increase the levels of IgE against rSm22.6 in IT/rSm22.6 group. If IgE against Sm22.6 is a key factor associated to resistance, the absence of protection observed in Sm22.6 immunized mice can also be related to the lack of Fc-ɛ receptors expression in murine eosinophils [33] and the use of other models is necessary in order to confirm the inability of rSm22.6 to induce protection in an animal previously sensitized by parasite antigens.

On the other hand, in Balb/c mice immunized with rSm29, we observed that the production of IgG, IgG1, IgG2a and IgE reaches the highest levels after the third immunization, which is consistent with the number of doses needed to confer protection against *S*. *mansoni* infection in animals previously exposed to parasite antigens. In individuals living in endemic areas for schistsosomiasis, the resistance to *S*. *mansoni* infection and reinfection is associated to increased production of IgG1 and IgG3 specific for Sm29 [11]. Regarding humoral profile the differences observed in antibody titers between rSm29 group, that did not develop a protective immunity and IT/rSm29 group, that developed a protective immunity, resides in an increased titer of IgG and decreased titer of IgG1. These results suggest that other IgG isotypes may be associated with the protection induced by this vaccine formulation.

Another concern regarding the antibodies produced after mice immunized with the recombinant proteins relied on their ability to recognize the native form of the protein on the parasite surface. In this context, the sera from Sm22.6 or Sm29 immunized mice recognized Sm22.6 and Sm29 expressed on the parasite surface, thus confirming the ability of the antibodies produced after immunization to bind the native form of the antigen.

In mice immunized with rSm22.6 a significantly increased production of IL-2, TNF-α, IFN-γ, IL-4, IL-10 and IL-17 was observed, demonstrating that this formulation induced a mixed cytokine profile, similar to that observed in naïve Balb/c mice or naïve C57BL/6 immunized with the same protein [9]. Despite high levels of inflammatory cytokines, significant amounts of IL-10 were also produced. IL-10 is an important regulatory cytokine which regulates the immune response triggered by *S*. *mansoni* infection [34,35,36,37]. Significant amounts of IL-10 were also observed in naïve mice immunized with rSm22.6 plus Alum or without adjuvant, which also did not confer protection to challenge infection [21]. Increased production of IL-10 was also associated with absence of protection in mice immunized with schistosomula tegument in the absence of adjuvant [38]. If IL-10 production is responsible to the lack of protection observed in rSm22.6 immunized mice still need to be determined.

In contrast to Cardoso and colleagues (2008), that observed a significant production of IFN-γ, TNF-α and IL-10 cytokine in C57BL/6 naïve mice immunized with the recombinant protein, in our protocol Balb/c immunization with the rSm29 triggered a significant production of IL-2, IFN-γ, IL-17 and IL-4 regardless of have been previous infected or not. Difference in cytokine profile between naïve Balb/c mice and infected/treated mice was only observed in IL-10 production that decreased in the infected/treated animals that presented a significant reduction in parasite burden.

An important player on the protective immunity induced by vaccines is memory cells [39]. Our study demonstrate that IT/rSm29 group present a significant increase in the percentage of TCD4 central memory cells in comparison with IT/Saline group and also significant increase in the percentage of TCD4 effector cells in comparison with Balb/c naïve mice immunized with rSm29. Since protection was observed only in the group of mice that had been previously infected with *S*. *mansoni* cercariae and treated with praziquantel, these cells might play an important role in parasite elimination.

Although helminth infections have been associated with impaired protective vaccine-induced immunity against heterologous pathogens [40,41,42,43,44], in our study a previous infection have contributed to vaccine-induced protection against schistososmiasis. Mice treatment before immunization schedule might have contributed to vaccination success either by enhancing the frequency of memory cells or decreasing IL-10 production in infected treated Sm29 immunized mice.

Different type of immune response can be triggered by different vaccine formulations, but their ability to induce protection depends, besides other factors, on the role of the target antigen on pathogen development and survival. In the case of schistososmiasis, for instance, different types of immune mechanisms against different targets have been associated with protection. In vaccine formulations using Sm28 (GST) as antigen, the protective immunity is related to activation of a robust humoral response that blocks antigen enzymatic activity impacting on worm burden and female fecundity [45,46]. In the other hand, protective immunity induced by rSm14 is dependent on IFN-γ and TNF-α production [47]. In the case of Sm22.6 and Sm29 the knowledge about their functions is still extremely scarce. Lin and He (2006) have suggested that the Sm22.6 has an important role in the regulation of human coagulation [48]. For Sm29, however, there is no information about its function. Functional analysis using RNA interference techniques (RNAi) can bring valuable information, allowing us to develop vaccine strategies that can effectively contribute to the parasite elimination [49].

In conclusion, ours results reinforces that the Sm29 is a promising vaccine candidate to compose an anti-schistosomiasis vaccine. Although Sm29 vaccination has failed to confer protection in Balb/c naïve mice, the fact that immunization of previously infected and treated animals trigger protection suggests that vaccination of individuals from endemic areas can be effective against *S*. *mansoni* infection, since it is a population sensitized by the parasite.

## Supporting Information

S1 Fig Frequency of activated monocytes in mice immunized with rSm29 or rSm22.6, or inoculated with saline. (TIF)
