# Supplementary figures and images for: Sm29, but Not Sm22.6 Retains its Ability to Induce a Protective Immune Response in Mice Previously Exposed to a Schistosoma mansoni Infection
Source: PLoS Negl Trop Dis. 2015 Feb 27;9(2):e0003537. doi: 10.1371/journal.pntd.0003537 (PMC4344193; doi:10.1371/journal.pntd.0003537)

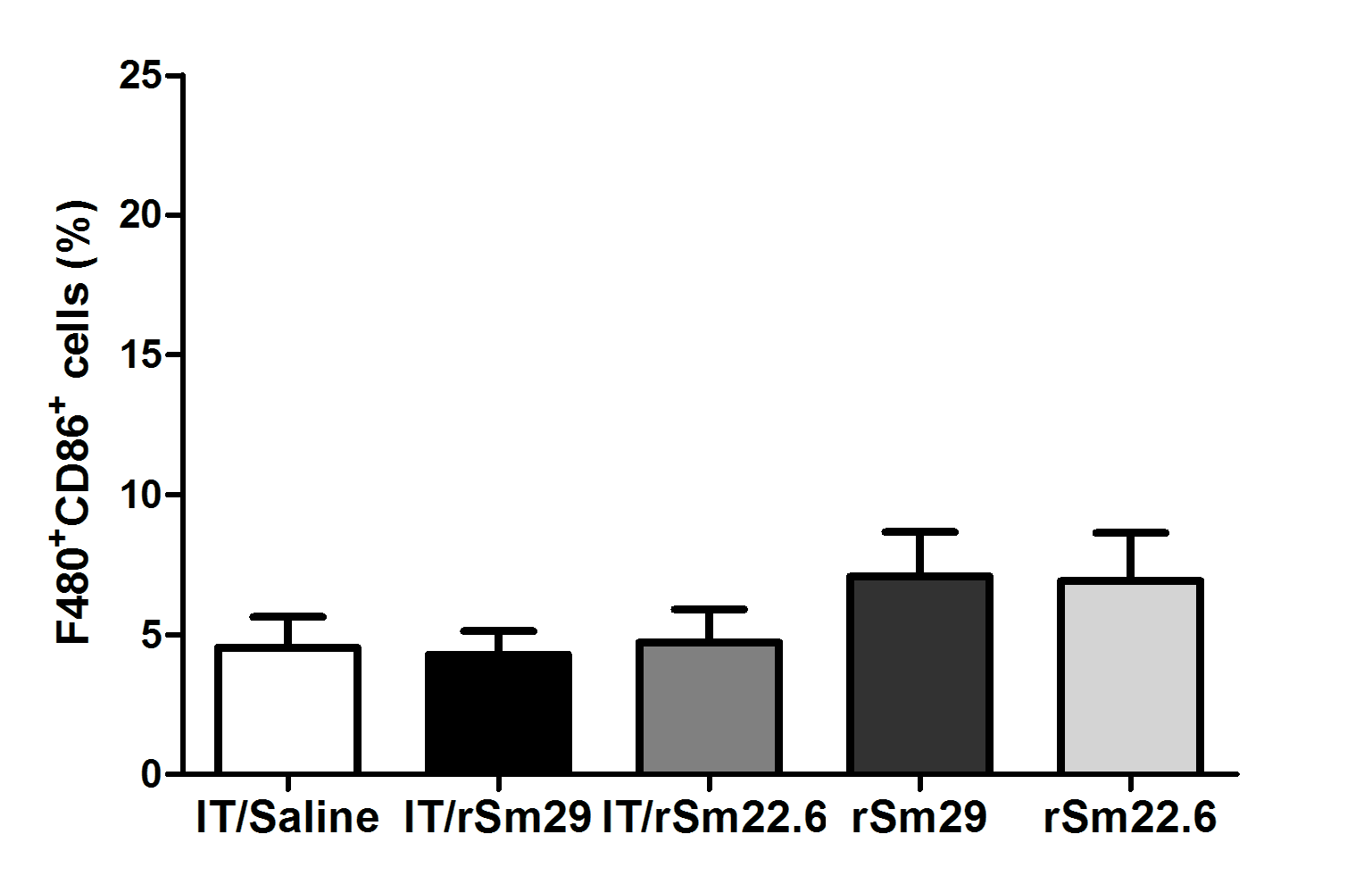

Supplement: S1 Fig — (TIF) [file pntd.0003537.s001.tif]
